# Investigations on the Impact of a Series of Alkoxysilane Precursors on the Structure, Morphology and Wettability of an Established Zirconium-Modified Hybrid Anticorrosion Sol–Gel Coating

**DOI:** 10.3390/gels10050315

**Published:** 2024-05-05

**Authors:** H. Alwael, E. MacHugh, M. S. El-Shahawi, M. Oubaha

**Affiliations:** 1 Department of Chemistry, Faculty of Science, King Abdulaziz University, P.O. Box 80203, Jeddah 21589, Saudi Arabia; hos351@hotmail.com (H.A.); mohammad_el_shahawi@yahoo.co.uk (M.S.E.-S.); 2 Centre for Research in Engineering Surface Technologies (CREST), FOCAS Institute, Technological University Dublin, 13 Camden Row, D08 CKP1 Dublin, Ireland; emh2435@gmail.com; 3 Detailine Ceramics Co., Ltd., 19 Castle Court, Killiney, A96 TY89 Dublin, Ireland

**Keywords:** alkoxysilanes precursors, hybrid sol–gel materials, structural characterisations, surface properties, wettability, coatings

## Abstract

The current study reports on the impact of a series of functional alkoxysilanes on the wettability and structure of a well-established silicon/zirconium hybrid anticorrosion sol–gel coating. The selected functional alkoxysilanes comprise tetra ethylorthosilicate (TEOS), 3-glycidyloxypropyltrimethoxysilane (GPTMS), 3-aminopropyltriethoxysilane (APTES) and vinyltriethoxysilane (VTES) and are incorporated at various concentrations (1, 5, 10 and 20%) within the silicon/zirconium sol–gel material. The prepared materials are successfully processed as coatings and cured at different temperatures in the range of 100–150 °C. The characterisation of the structures and surfaces is performed by dynamic light scattering (DLS), scanning electron microscopy (SEM), Fourier transform infrared spectroscopy (FTIR), silicon nuclear magnetic resonance spectroscopy (^29^Si-NMR), atomic force microscopy (AFM) and static water contact angle (WCA). Structural characterisations (DLS, FTIR,^29^Si-NMR) show that the functional alkoxysilanes effectively bind at the surface of the reference sol–gel material, resulting in the formation of functional core–shell nanoparticles. WCA results show that the hydrophobic properties of all materials decrease with curing temperature, and AFM analysis demonstrated that this behaviour is associated with a decrease in roughness. The physico-chemical processes taking place are critically assigned and discussed.

## 1. Introduction

Nowadays, there is an ever-increasing request for the development of innovative multifunctional hybrid materials for a wide range of applications, including optical devices [1,2,3,4,5], (bio)sensors [6,7,8] and protective coatings [9,10,11]. Thanks to the unique combination of organic and inorganic compounds, hybrid materials offer unlimited possibilities for the development of multifunctional materials [12]. For example, the fabrication of complex optical devices such as miniature 3D photonic crystals simultaneously requiring high resolution, controllable refractive indexes and mechanical stability has become possible by two-photon polymerisation of hybrid sol–gel materials [13], which has enabled the development of 3D miniature medical devices [14]. To date, of the various preparation methods, the sol–gel process is probably the most popular route for the preparation of hybrid materials [12]. The process consists of hydrolysis and condensation reactions of alkoxide precursors, with the most widely used alkoxide precursors being the alkoxysilanes, which can be used separately or in combination to achieve nanostructured hybrid materials with mesoporous morphologies [12].

For anticorrosion application, in addition to the chemical resistance, mechanical strength and adhesion to metal substrates, coatings require hydrophobicity to reduce interactions between the metal surface and corrosive agents, usually entrapped in water [15,16,17]. Amongst the numerous anticorrosion sol–gel coatings reported in the state of the art, a formulation based on the combination of 3-trimethoxysilylpropylmethacrylate and a zirconium complex has been intensively investigated and has shown exceptional performances for the protection of aluminium alloys against corrosion [18,19,20,21,22,23,24,25,26,27]. Different strategies have been employed to improve the anticorrosion barrier properties of this formulation, including varying the ratio of the Si/Zr [18,19], the hydrolysis conditions [20], the nature of the chelating agent [21,22], the incorporation of anticorrosion compounds [23], and silanes such as TEOS [24,26] and diphenylsilanediol [27].

To further advance the investigations of this well-established material, we propose altering the wettability of the coatings by incorporating additional alkoxysilane precursors. For this purpose, four hybrid alkoxysilane precursors containing different organic functionalities were selected. These include tetraethylorthosilicate (TEOS), 3-glycidyloxypropyl-trimethoxysilane (GPTMS), 3-aminopropyltriethoxysilane (APTES) and vinyltriethoxysilane (VTES). While those precursors all have the potential to react with the reference material via their alkoxysilane counterpart, they differ in the nature of the pendent functional group (epoxy, amine and vinyl for GPTMS, APTES and VTES, respectively). This may alter the surface properties of the initial nanoparticles and their condensation process during the formation of the coating. Therefore, if effective binding at the surface of the sol–gel nanoparticles takes place, functional core–shell nanoparticles [28] can be formed with possible effects on the structural and wetting properties of the final coatings. The structure of the materials was investigated via particle size analysis using dynamic light scattering (DLS), Fourier transform infrared spectroscopy (FTIR), and silicon nuclear magnetic resonance spectroscopy (^29^Si-NMR), while the wettability of the prepared coatings will be determined using water contact angle (WCA).

Thus, the current study focuses on the following: (i) preparing a series of functional hybrid materials based on the introduction of different alkoxysilane precursors (TEOS, GPTMS, APTES and VTES) within a reference Si/Zr sol–gel material; (ii) characterising the structures and surface properties of the developed materials; (iii) discussing the role of the alkoxysilane functional groups on the observed structures and properties.

## 2. Results and Discussion

### 2.1. Particle Size Analysis

**Figure 1:**
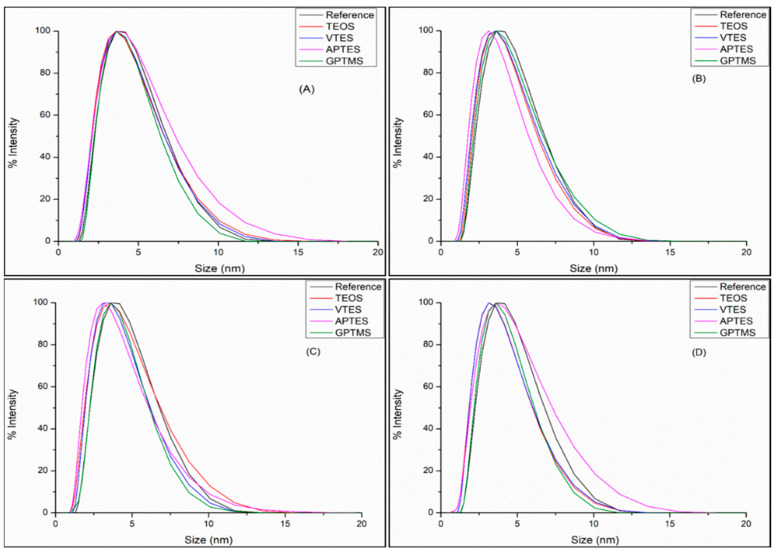
DLS spectra of the four functional silanes-modified materials after 24 h aging at 1.0 (**A**), 5.0 (**B**), 10 (**C**) and 20% (**D**) concentration.

**Figure 2:**
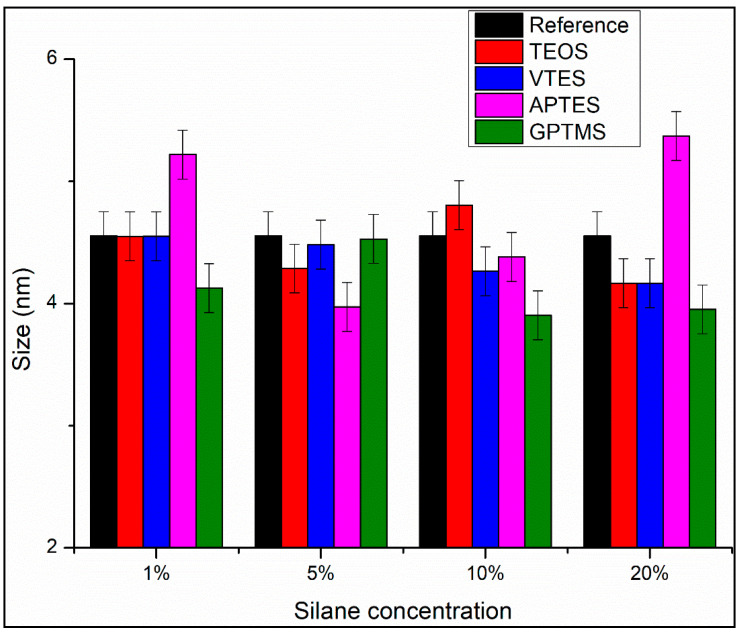
Full width at half maximum (FWHM) for all functional silanes modified materials.

The particle sizes of the sols were recorded 24 h after preparation, as displayed in Figure 1. All spectra revealed a single distribution band in the 0–20 nm range that is centred at 3.6 nm (±1 nm), regardless of the concentration of the functional alkoxysilane. On the other hand, the reference material modified with 1% of functional silanes displayed no variation in the maximum nanoparticle size, whereas, for the other concentrations, the maximum particle size fluctuates around 3.6 nm within the error bar of 1 nm (Figure S1). Thus, it can be concluded that the addition of the functional silane does not significantly alter the particle size of the materials. In addition, the values of the full width at half maximum (FWHM) were measured (Figure 2) and showed that they all range within 1.5 nm (from 4 to 5.5 nm). Nevertheless, at this stage, it is worth noting that, although not significantly obvious, the DLS results tend to show that APTES exhibits a different reactivity towards the reference material. Indeed, the minor decrease in the maximum nanoparticle size for samples containing 5 and 10% APTES and the sudden increase for a concentration of 20% along with a similar variation in the FWHM is an indication that the APTES concentration may play a role in the structure of the reference material, probably due to its double functionality (amine and alkoxysilane groups). To explain this behaviour, one can speculate that for the concentrations below 20%, the APTES tend to form a shield around the nanoparticles of the reference materials, which would aggregate the smallest nanoparticles, as the maximum nanoparticle size is slightly decreased and the distribution band tending to shift towards smaller sizes. For a 20% APTES concentration, it is possible that the aggregation process of larger particles takes place, thus explaining the minor increase in the maximum nanoparticle sizes and FWHM as well as the enlargement of the distribution towards larger sizes. These assumptions are further discussed below in the FTIR study (Section 2.3). Therefore, based on the DLS data, it can be concluded that no significant alteration of the nanoparticle distribution is evident, with the exception of the material functionalised with APTES.

### 2.2. Water Contact Angle (WCA)

**Figure 3:**
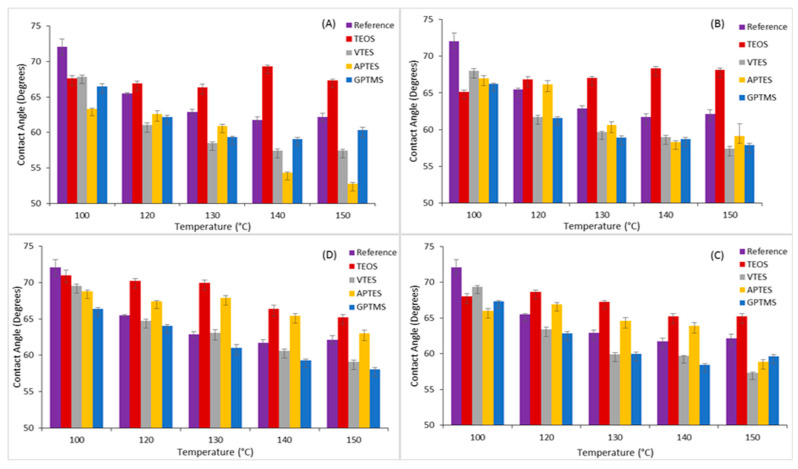
WCA measurements vs. temperature for all materials at 1.0 (**A**), 5.0 (**B**), 10.0 (**C**,**D**) and 20.0% (**D**) concentrations of the functional alkoxysilane precursors.

**Scheme 1:**
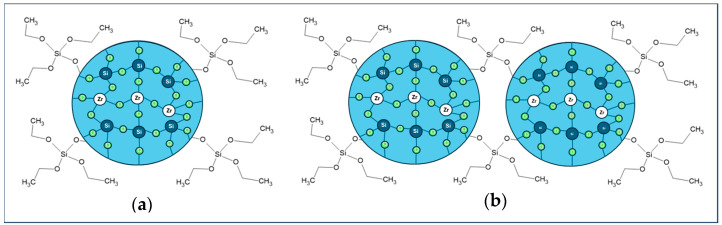
A schematic representation of the impact of TEOS at the surface of sol–gel nanoparticles before curing (**a**) and after curing (**b**).

WCA measurements were performed, and the average values for all materials at a given concentration are illustrated in Figure 3. It can be observed that for the reference material, the WCA values decrease by 10° from 72 to 62° when the curing temperature increases from 100 to 130 °C and is stable up to 150 °C. Unlike the reference material, the TEOS-modified coatings show WCAs in the range of 65–69°, regardless of the curing temperature and TEOS concentration. Of note, a slight tendency to WCA decrease at high TEOS concentrations is observed within this range at concentrations of 10 and 20%. Therefore, the wettability of the materials modified with TEOS appears to be less impacted by the curing temperature than those observed for the reference material. Thus, it can be anticipated that TEOS enables the maintenance of most of the hydrophobic siloxane groups formed during the initial curing process, as sketched in Scheme 1.

**Scheme 2:**
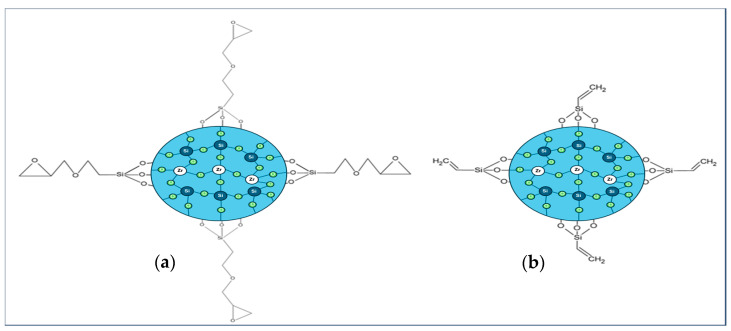
A schematic representation of the effect of GPTMS (**a**) and VTES (**b**) on the formation of stable sol–gel nanoparticles.

Regardless of the used concentration, GPTMS and VTES showed a similar trend to the reference coating with a progressive decrease in the WCAs from 100 to 130 °C and a quasi-stabilisation up to 150 °C. However, the reference coating shows higher WACs than those recorded for GPTMS and VTES, indicating that GPTMS and VTES would form a core around the nanoparticles that reduce their condensation ability to form hydrophobic siloxane groups, as shown in Scheme 2.

**Scheme 3:**
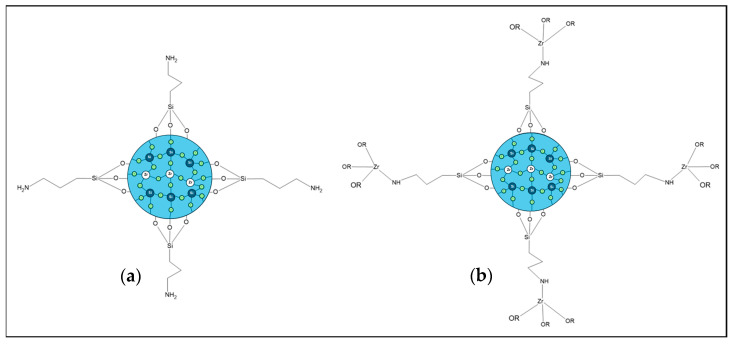
A schematic representation of the possible reaction of APTES with the reference material to form pendant amine groups (**a**) or transition metal complexes (**b**).

Similarly to the reference coating and the coatings modified with GPTMS and VTES, at concentrations of up to 5%, APTES experienced a regular WCA decrease with the increase in the curing temperature. However, for concentrations of 10 and 20%, a hydrophobic recovery is clearly observed, regardless of the curing temperature. This indicates that competition between the formation of hydrophilic and hydrophobic takes place as a function of the APTES concentration. Indeed, one can anticipate that below 10%, APTES may bind to the surface of the reference material via its ethoxide groups, thus leaving intact its hydrophilic amine groups at the surface (Scheme 3a). For the concentrations of APTES greater than 5%, the formation of hydrophobic species such as siloxane and silicon–zirconium oxide groups may be preferably favoured. Indeed, unlike TEOS, GPTMS and VTES, APTES contains an amine group that may catalyse the condensation process silanol and zirconium hydroxide group into siloxane and silicon–zirconium oxide groups but also react with the zirconium atom to form complexes (Scheme 3b). FTIR results were undertaken to clarify this question and better understand the physico-chemical process taking place.

### 2.3. FTIR Spectral Measurements

**Figure 4:**
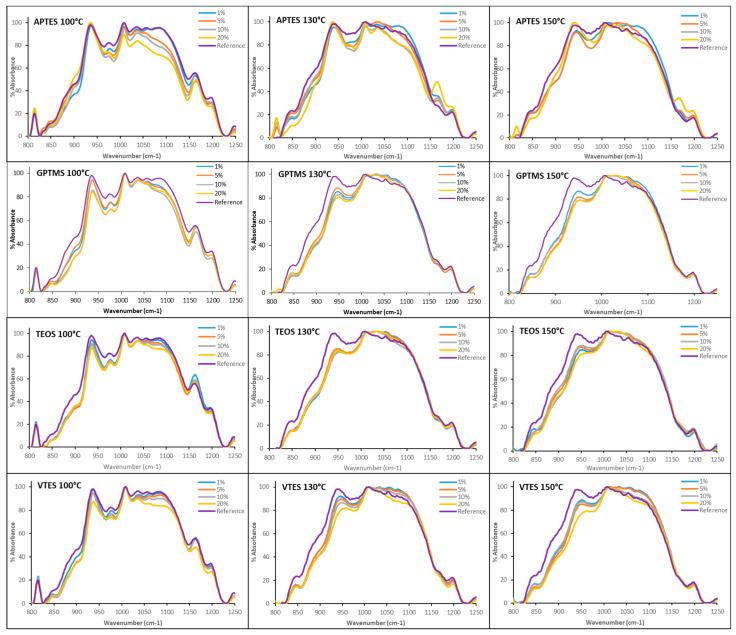
FTIR spectra of the synthesised sol–gel materials.

To properly identify the impact of the chemical alterations and curing processes on the structure of the materials, the FTIR spectra (800–1250 cm^−1^) of all materials were recorded (Figure 4). All spectra displayed the characteristic vibrations of Si-O-C (810 and 1170 cm^−1^), Si-OH (840 cm^−1^), Si-O-Si (880 and 1000–1100 cm^−1^) and Si-O-Zr (940 cm^−1^) in the reference materials [29,30,31]. The large band located in the 1000–1100 cm^−1^ spectral range was safely assigned to the asymmetric siloxane vibrations, where in this region, the four superimposed bands located at 1010, 1030, 1055 and 1090 cm^−1^ were identified in purely inorganic silicate materials [29,30,31]. Those bands were also identified in similar hybrid sol–gel materials prepared from combinations of zirconium complexes and single or multiple hybrid alkoxysilanes, including MAPTMS and TEOS [32], GPTMS [33], GPTMS and TEOS [34], octlytrimethoxysilane [35], VTES and methyltrimethoxysilane [36]. These studies fully support our above assignments.

The band located at 1090 cm^−1^ was attributed to the stretching vibration of the Si-O-Si groups located at the interface between tetrahedral and octahedral units (called silicon apical oxide units), whereas the other three bands at 1010, 1030 and 1055 cm^−1^ were safely assigned to stretching vibrations of siloxane bonds within symmetrical tetrahedral units [29,30,31]. Thus, the observed vibration located at 1090 cm^−1^ (Figure 4) represents the formation of high-energy siloxane bonds originating from the aggregation of nanostructures of different sizes and possibly different densities, while bands located at 1010, 1030 and 1055 cm^−1^ represent siloxane bonds within networks of lower connectivity. It is worth mentioning that, as the wavenumber of the vibration increases, the energy of the bond also increases, symbolising a more connected network.

#### 2.3.1. Analysis at 100 °C

**Scheme 4:**
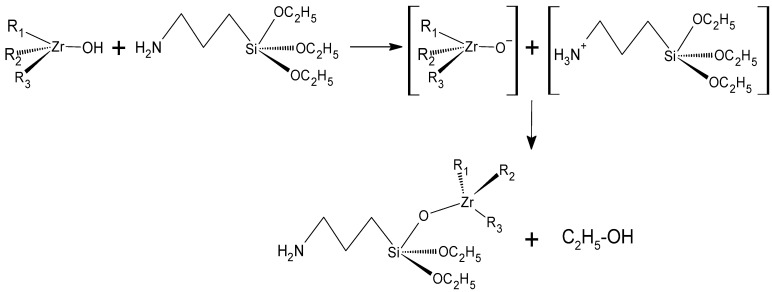
A schematic diagram describing the reaction of APTES with Zr-OH groups.

Except for APTES, all modifiers provoke a decrease in the Si-O-Zr bond (940 cm^−1^). All materials, except APTES at 1%, show a decrease in the high-energy siloxane (1090 cm^−1^) and low-energy (1010 cm^−1^) bonds. In parallel, no significant change in the Si-O-C bond (810 and 1170 cm^−1^) is observed for all materials, but a strong decrease in the Si-OH bonds (840 cm^−1^) is noted. This indicates that at 100 °C, all materials favourably react with the residual Si-OH groups of the reference material, forming preferably core–shell nanoparticles, supporting our initial hypothesis in Section 2.2. Because the intensity of the Si-O-Zr bond is stable in the APTES-modified materials and the Si-O-Si bonds are decreasing, it indicates that APTES exhibits a greater reactivity with the residual Zr-OH (and possibly Zr-O-C_3_H_7_) groups than the three other alkoxysilanes. This can only be explained by the ability of the amine group to catalyse the condensation reactions of alkoxysilanes [31,37], thus the formation of Si-O-Zr bonds, as proposed in Scheme 4.

#### 2.3.2. Analysis at 130 °C and 150 °C

The increase in the curing temperature provokes an increase in the intensity of all siloxane bonds, translating the condensation of the residual silanol and un-hydrolysed alkoxysilane groups. In parallel, except for the materials containing APTES, for all other modifiers, the Si-O-C bonds (810 and 1170 cm^−1^) have dramatically decreased at 130 °C and fully disappeared at 150 °C, suggesting their full condensation. Unlike the other material, these results confirm that APTES forms preferably Si-O-Zr groups with the mechanism proposed in Scheme 4 above. In addition, for the GPTMS-, TEOS- and VTES-modified coatings, it can be seen that the intensity of the Si-O-Zr bond (940 cm^−1^) also increased and generally overtook the reference material. This behaviour is attributed to the thermal activation of the condensation reactions of the residual un-hydrolysed alkoxide groups together or with the residual uncondensed silanol and zirconium hydroxide groups.

Owing to these results, one can conclude that at low temperatures, the coatings are formed of pendant functional groups that reduce the condensation of the reference material, likely leading to coatings with higher porosity and roughness. The increase in the curing temperature provokes an increase in the condensation of the reference material, likely leading to denser coatings with a lesser roughness. Nevertheless, the FTIR results would suggest that the hydrophobic properties should increase for all materials as a function of the curing temperature. However, the opposite trend is shown in the WCA results. This requires AFM analysis to clarify this point.

### 2.4. AFM Analysis

**Figure 5:**
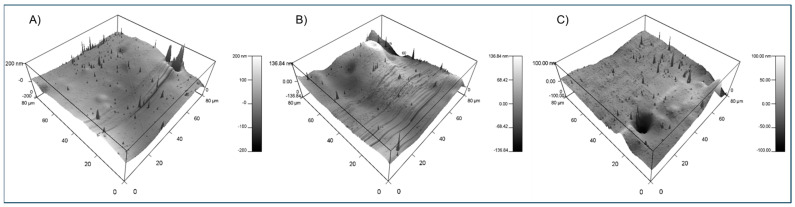
AFM analysis of the 5% GPTMS modified coatings cued at 100 °C (**A**), 130 °C (**B**) and 150 °C (**C**).

AFM analysis was performed to identify if the curing process within the investigated range of temperature could alter the morphology of the coatings. Typical recorded AFM images are presented in Figure 5 (an example of the 5% GPTMS-modified material). AFM images of other 5% modified samples are available in the Electronic Supplementary Information (Figure S2). The roughness of the coatings is measured from the AFM images and is presented in Table 1. The black holes on some of the images are certainly due to impurities originating from the ambient atmosphere, although care was taken to minimise contamination during the processing and deposition of the coatings. Surprisingly, some irregular peaks with heights of several tens of nm are seen at the surface of some coatings, which can be due to a vertical growth of the sol–gel probably initiated by the solvent evaporation phase. It is difficult, however, to identify if those are impacted by the curing temperature. The measured roughness values of the coatings show a decrease of 50% from 100 °C to 150 °C, suggesting that the coatings undergo a levelling process definitely due to the condensation of the material, as shown by FTIR results. Consequently, by corroborating the FTIR analysis with the AFM results, it may be concluded that the decrease in the roughness due to the condensation process could be the prevalent factor observed for this series of materials. These conclusions are in agreement with a number of studies summarised in an excellent review article in the field [38].

### 2.5. ^29^Si-NMR

**Figure 6:**
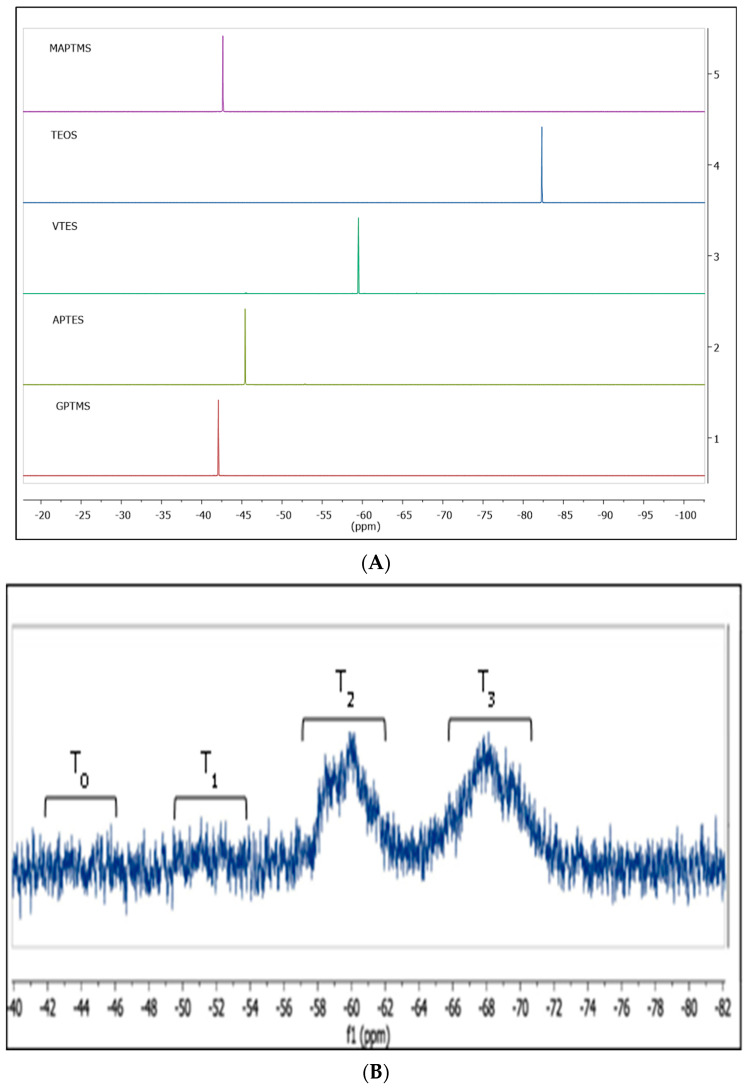
^29^Si-NMR spectra of pure four alkoxysilane precursors (**A**) and the reference material (**B**).

^29^Si-NMR spectra of the pure functional alkoxysilanes were initially recorded to characterise the purity and chemical shifts before the reaction with the reference sol–gel material, as shown in Figure 6A. A well-defined and sharp single peak was easily observed at −42.6, −82.5, −59.5, −45.4 and −42.4 ppm for MAPTMS, TEOS, VTES, APTES and GPTMS, respectively. This translates to high-purity precursors with no hydrolysed species.

**Figure 7:**
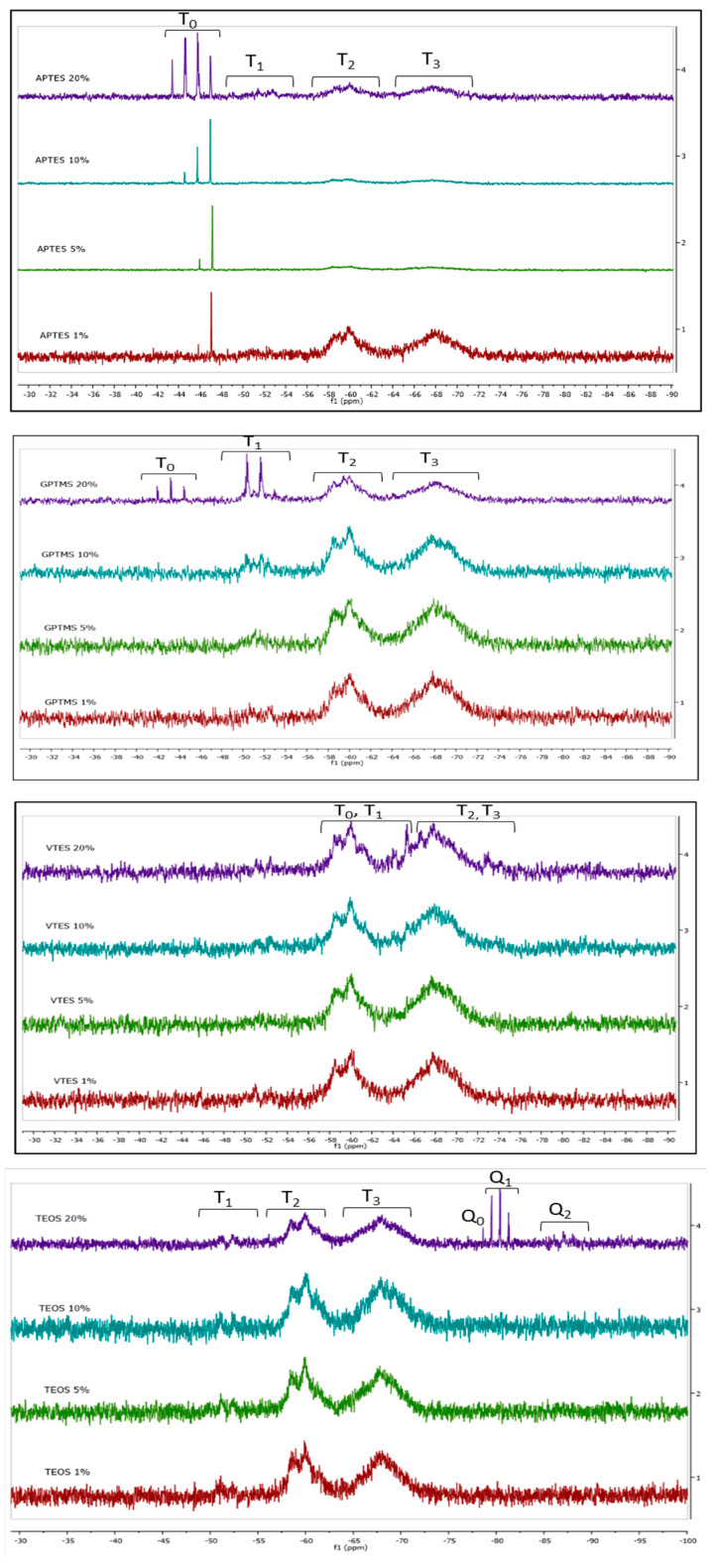
^29^Si-NMR spectra for the synthesised four sol–gel materials.

The ^29^Si-NMR spectrum of the reference sol–gel material is shown in Figure 6B, and ^29^Si-NMR spectra for all materials are shown in Figure 7. T_n_ and Q_n_ notations are used to identify the number of Si-O-Si units in tri-alkoxysilanes and tetra-alkoxysilanes-based materials, respectively. The subscript n represents the number of connected Si-O-Si units.

**Figure 8:**
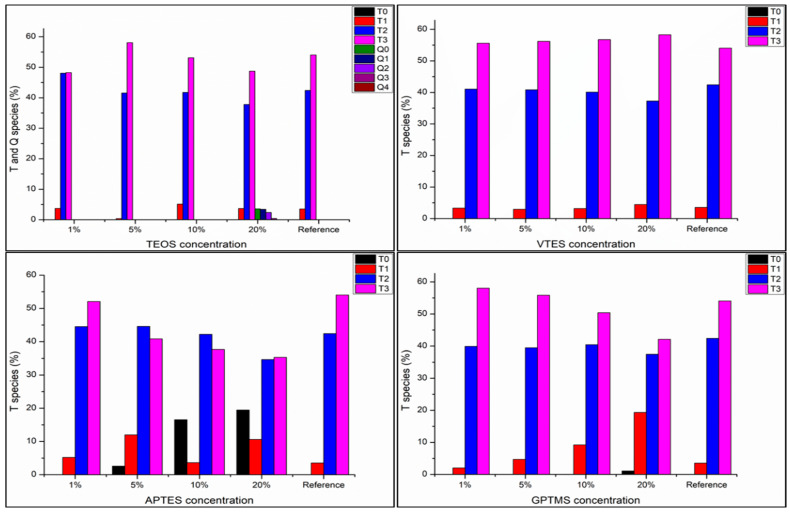
T and Q species concentration for all materials.

The content of T_n_ and Q_n_ species within each material is presented in Figure 8. The reference sol–gel material contains no T_0_, 3% of T_1_, 42.5% of T_2,_ and 54.5% of T_3_ species. Thus, any T_0_ signals in the ^29^Si-NMR spectra (Figure 7) can only be attributed to the alkoxysilanes precursors added to the reference material. For better clarity, the NMR results for each of the materials are analysed separately in the following sub-sections.

#### 2.5.1. Material modification by APTES

**Scheme 5:**
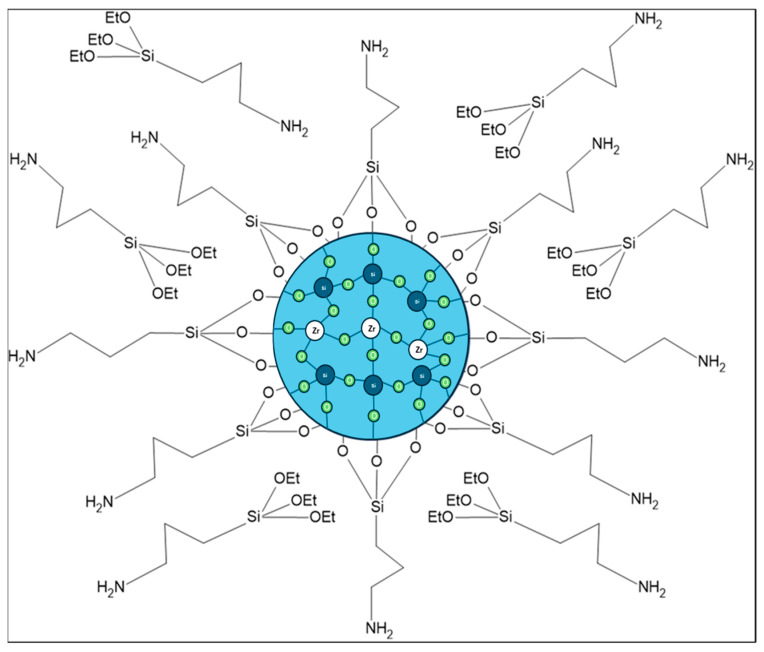
A schematic representation of saturated reference sol–gel nanoparticle surrounded by unbound APTES.

The characteristics signal of the pure precursor at −45.4 ppm was found absent at all concentrations of the materials modified with APTES, suggesting the full reaction of the APTES with the sol–gel material. At concentrations of 1 and 5%, two singlet peaks located at −45.9 and −47.0 ppm were observed. These two signals are located at higher chemical shifts than the one of the precursor, indicating that the electronic environment of the silicon is increased. The chemical shift located at −47.0 ppm is attributed to T_1_ species, whereas the peak found at −45.9 was safely assigned to T_0_ species where the silicon is an obstructed environment [38]. The saturation of the surface of the reference sol–gel nanoparticles by APTES molecules, leading to unbound but closely entrapped molecules, is more likely to account for this trend, as sketched in Scheme 5. For the 10% APTES sample, an additional signal at −44.6 ppm is observed, which is located at a lower chemical shift than the pure APTES precursor (−45.4 ppm), indicating that the electronic environment of the silicon atom has decreased and that this signal can only be recognised as T_0_ species in a less obstructed environment probably associated with the substitution of an ethoxy group by a hydroxyl group (Si-OH) [39].

At a concentration of 20% of APTES, an additional signal is observed at −43.4 ppm, and the duplication of the chemical shift at −44.6 ppm into two doublets was observed, confirming the de-shielding effect observed at the 5% sample. Thus, the additional signal located at −44.6 ppm is safely attributed to the T_0_ species with two silanol groups Si(OH)_2_, while the signal at −43.4 ppm would translate the formation of T_0_ species with three silanol groups Si(OH)_3_. Thus, it can be concluded that APTES is effectively covalently bound to the surface of the reference sol–gel nanoparticle, and at 1% APTES concentration, it fully saturates the surface of the nanoparticles of the reference sol–gel material. The extra addition of APTES provokes the formation of free hydrolysed APTES molecules.

#### 2.5.2. Material Modification by GPTMS

In all spectra of the pure GPTMS precursor, the signal is absent, suggesting its interaction or reaction with the sol–gel matrix. The appearance of the T_1_ species between −50 and −52 ppm, and their growth (2.0, 4.0, 9.0 and 19.0%), as the content of GPTMS increases (1.0, 5.0, 10 and 20.0%), represents the main difference with the NMR spectrum of the sol–gel matrix. Thus, GPTMS is fully bound to the surface of the sol–gel matrix, and at high GPTMS concentration, T_0_ species appeared at −41.9, −43.2 and −44.9 ppm, signifying the presence of free GPTMS molecules in a less obstructed environment. Similarly to the APTES, the explanation for this phenomenon is the hydrolysis at different degrees involving Si(OH)_1_, Si(OH)_2_ and Si(OH)_3_, signifying that the GPTMS can be fully bound at concentrations up to 10% and that the additional GPTMS molecules are present as free hydrolysed molecules within the overall system. In comparison with APTES, GPTMS has the ability to link with the sol–gel matrix from 10% concentration, whereas the APTES can bind with the sol–gel matrix even at 1% concentration of free APTES; hence, it can be postulated that the APTES would form an electron-rich shell around the sol–gel matrix via the free pair of electrons on the N atom of the amino group, thus provoking electronic repulsion of additional APTES molecules leaving them as free and unreacted hydrolysed molecules.

#### 2.5.3. Material Modification by VTES

Regardless of the concentration, the addition of VTES precursor into the sol–gel matrix displayed a peak at −60 ppm, related to the T_0_ species of the VTES precursor. The condensed species of the VTES overlap with the T_3_ band of the sol–gel, and at low concentrations, they cannot be distinguished. However, with the addition of 10 and 20% concentrations of VTES into the sol–gel matrix, three bands at −65.3, −66.8 and −73 ppm are observed, signifying the condensation of the VTES precursor with the reference sol–gel. These bands are safely assigned to T_1_, T_2_ and T_3_ groups within the VTES organosilane, respectively, supporting functionalisation of the surface of the reference matrix with VTES.

#### 2.5.4. Material Modification by TEOS

Similarly to GPTMS, Q_o_ signals are present at a concentration of 3.5% of the precursor for the highest concentration of TEOS. The four signals observed at −78.61, −79.50, −80.41 and −81.33 ppm are most likely corresponding to various hydrolysed species of TEOS that do not contain any bridging Si-O-Si groups [29]. Importantly, at concentrations of 3.4 and 2.3% TEOS, Q_1_ and Q_2_ species were observed, whereas Q_3_ species were not clearly detected at 0.4%. These species correspond to mono-, di- and tri-substituted bridging Si-O groups [29] and suggest the binding of TEOS with the reference sol–gel nanoparticles. Thus, the functional alkoxysilanes are effectively bound to the reference material, enabling the formation of core–shell nanoparticles and confirming the suggested hypothesis on the formation of surface functionalised nanoparticles and coatings.

### 2.6. SEM Analysis

**Figure 9:**
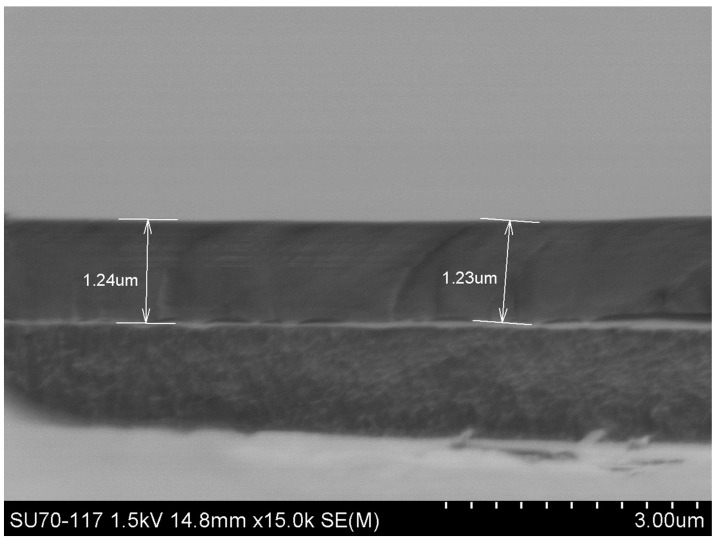
SEM image of the cross-section of hybrid sol–gel.

SEM analyses were performed on coatings deposited on AA2024-T3 aluminium substrates. All coatings showed similar structures and were found to be continuous and homogeneously deposited on aluminium substrates with a coating close to 1.2 micron ± 0.25 micron. The thickness fluctuation is due to the likely alteration of the viscosity of the sols. A representative SEM image is shown in Figure 9 (of the reference sol–gel material containing 1% APTES).

## 3. Conclusions

In the search for strategies to increase the hydrophobic properties of an established silicon/zirconium hybrid anticorrosion sol–gel coating, APTES, GPTMS, TEOS, and VTES were selected thanks to their potential hydrophobic functional groups. Those alkoxysilane modifiers were successfully incorporated within the reference coating, and structural characterisations were undertaken. FTIR and ^29^Si-NMR results concord to show that surface functionalisation of the reference material is effective by the formation of core–shell nanoparticles and that the condensation process of the inorganic network relies on the applied curing temperature. The WCA values were seen to decrease with the curing temperature, while the formation of hydrophobic species was identified by FTIR. AFM results showed that this behavior can be attributed to the decrease in surface roughness. Although the achieved WCA values were found to range in the hydrophilic domain, it appears that TEOS would be the most suitable modifier to maintain the wetting properties of the reference material, especially for coatings exposed at temperatures greater than 100 °C.

## 4. Materials and Methods

### 4.1. Preparation of the Hybrid Sol–Gel

Preparation of the reference hybrid sol–gel material was reported by MacHugh et al., 2019 [29], and the experimental steps are depicted in the Electronic Supplementary Information (Figure S3). This scheme employs a four-step sol–gel process as follows: Step 1: 3-trimethoxysilylpropylmethacrylate (MAPTMS) is hydrolysed employing an aqueous solution of HNO_3_ (0.1 M) at a 25% hydrolysis rate vs. the reactive alkoxysilane precursors for 45 min, while zirconium (IV) propoxide (ZPO) is complexed with methacrylic acid (MAAH) in equimolar conditions for the same duration; Step 2: the mixture of the two solutions is stirred for a period of 10 min; Step 3: the mixture is hydrolysed with deionised water to achieve a 50% hydrolysis rate of the total alkoxide groups and left to stir for 24 h. The final molar composition of the reactive precursors is MAPTMS/ZPO/MAAH: 2.5/1/1. Step 4: the mother batch of the synthesised reference material, as described above, is then divided into 4 equal fraction volumes in four separate beakers to which the functional alkoxysilanes are added.

The concentration of the various alkoxysilanes is 1, 5, 10 and 20 mol.% against the methoxy groups of the MAPTMS, as detailed in Table S1. Each concentration of alkoxysilane was added to an equal molar quantity of matter under constant stirring and under ambient conditions, and the prepared materials were finally stirred for 24 h before use. All chemicals used here are detailed in Table 2.

### 4.2. Coatings Preparation

The coatings were prepared in a clean-room environment to achieve particle-free coatings on glass substrates using the WS-650-23B spin-coater from Laurell. With this spin-coater, coating deposition can be performed by a multi-step deposition process in a precisely controlled atmosphere. The glass substrates were initially sonicated and rinsed in isopropyl alcohol (IPA) and dried in an oven to ensure no humidity or particles were present on the glass surface. Prior to deposition, the spin-coater chamber was fully saturated with IPA to control the evaporation of the solvents and minimise this effect on the surface roughness. The sols were initially filtered using a 0.45 µm syringe filter and processed by spin-coating, employing the following three-step deposition process: Step 1—deposition of the sols: the sols are deposited, and the substrate is immediately rotated at 200 rpm to ensure a slow and full coverage of the glass substrate. Step 2—thickness adjustment: the speed of the spin-coater is increased to 900 rpm for 35 s. Step 3—solvent evaporation: a small circular slot of 1 cm of diameter located on the spin-coater lid is opened, and the speed of the spin-coater is decreased to 250 rpm for 90 s to enable a slow removal of the solvent and achieve coatings with optical quality. Following deposition, the coatings were left to dry at ambient temperature for 1 h before undergoing the final curing for 1 h at various temperatures in the range of 100–150 °C, using a Binder drying oven (FD series).

### 4.3. Characterisation

#### 4.3.1. DLS

A Malvern Nano Zs instrument was used to determine the particle size of the materials. To avoid scattering and to achieve a system where particles are in a quasi-isolated environment, the sols were diluted in IPA in a ratio of 95:5% *v*/*v*, IPA: sol and filtered using a 0.45 µm Whatman syringe filter prior to each run. All sol–gel materials were prepared 5 times during a period of a week, and DLS spectra were recorded 24 h after synthesis completion. For reproducibility and statistical analysis, the prepared samples were run 5 times, and the presented spectra are the average of the 5 recorded spectra. The measurement uncertainty on the DLS instrument is 1 nm.

#### 4.3.2. Contact Angle Measurements

In this experiment, the static sessile drop method was used to measure the WCAs of the coatings using deionised water under ambient conditions. An electronic syringe and an FTA200 (First Ten Angstroms goniometer, software 2.0, Newark CA 64560, USA) were used to drop 5 microliters of deionised water onto the surface of the coatings, which were allowed to stabilise for 10 s before recording the digital images of the droplets. The images were then analysed using FTA32 Video 2.0 software, where 6 contact angle measurements were taken for each sample, and the average measurement was calculated as average ± standard deviation.

#### 4.3.3. FTIR

A Perkin Elmer GX instrument FTIR (4000–650 cm^−1^) was used for recording the FTIR spectra (4000–650 cm^−1^) in the reflection ATR configuration to allocate the different vibrational modes of the chemical species and also to assign the impact of the functional silanes on the structure of the reference sol–gel material. To enable a proper comparison of the spectra in terms of materials sol–gel reactions and condensations of the reactive silanes and zirconium species, all recorded spectra were normalised in the range 800–1225 cm^−1^ using the OriginLab 2024 software.

#### 4.3.4. AFM

A scanning AFM (Pacific Nanotechnology: Nano–RTM, Santa Clara, CA, USA) was used to characterise the surface topography and roughness of the coatings. At least three measurements were taken of the same scan size of 80 × 80 µm from three different areas of the sample surface. The root mean square roughness was measured, and the average value of the surface roughness was calculated.

#### 4.3.5. ^29^Si-NMR

A Bruker Avance II 400 MHz spectrometer was used to record the ^29^Si-NMR spectra to identify the chemical reactions between the silane modifiers and reference sol–gel at room temperature. An INEPTRD pulse sequence was used to record the NMR spectra using deuterated dimethylsulfoxide (DMSO) as a lock solvent.

#### 4.3.6. SEM

SEM images were performed on the cross-section of the coatings deposited on aluminium surfaces to identify their thickness, homogeneity and adhesion. The SEM images were recorded employing a Hitachi SU-70 SEM with an electron energy of 2–10 keV. Prior to analysis, the samples were sputter coated with a palladium layer of approximately 6 nm in thickness using a Cressington 208HR sputter coater.

## Figures and Tables

**Table 1:** Roughness values for the 5% GPTMS coatings as a function of curing temperature.

Sample	Curing Temperature (°C)	Surface Roughness (nm)	Error Bar (nm)
APTES 5%	100	120	±10
	130	92	±10
	150	84	±5
GPTMS 5%	100	80	±10
	130	52	±5
	150	45	±5
TEOS 5%	100	100	±10
	130	85	±5
	150	65	±5
VTES 5%	100	68	±10
	130	57	±5
	150	35	±5

**Table 2:** Origin and purity of the employed chemicals.

Chemical Name	Acronym	Supplier	Purity
3-trimethoxypropyltrimethoxysilane	MAPTMS	Safic-Alcan	98
Zirconium n-propoxide	ZPO	ABCR	70
Methacylic acid	MAAH	Merck	99
Tetraethyl Orthosilicate	TEOS	Merck	98
Vinyltriethoxysilane	VTES	Merck	97
3-Glycidyloxypropyltrimethoxysilane	GPTMS	Merck	98
3-Aminopropyltriethoxysilane	APTES	Merck	99

## Data Availability

The current works extend the impact of impregnation of a series of functional alkoxysilanes individually on the structure, wettability and curing temperature of the reference sol–gel material towards coatings. A proposed mechanism of the variation in the WCA values is associated with the reactivity of the functional silanes, primarily with the reference sol–gel matrix. Other functional alkoxysilanes and their combinations achieve greater performances along with the employment of fine structural characterisation techniques to afford more fundamental information on the mechanism of formation of nanostructures.

## Supplementary Materials

The following supporting information can be downloaded at: https://www.mdpi.com/article/10.3390/gels10050315/s1, Figure S1: Variation of the maximum particle size versus the concentration of silane modifier. Figure S2: AFM images of 5% silane modified coatings cured at 100, 130 and 150 °C. Figure S3: A scheme describing the four steps of the preparation of the reference hybrid sol-gel material by MacHugh et al., 2019 [29]. Table S1: Material formulations for functionalized alkoxysilanes at various concentrations (1.0–20 mol. %) against the methoxy groups of the MAPTMS.
