# Supplementary material for: Investigations on the Impact of a Series of Alkoxysilane Precursors on the Structure, Morphology and Wettability of an Established Zirconium-Modified Hybrid Anticorrosion Sol–Gel Coating
Source: Gels. 2024 May 5;10(5):315. doi: 10.3390/gels10050315 (PMC11121259; doi:10.3390/gels10050315)
Supplement: Supplementary file 1 [file gels-10-00315-s001.zip › gels-2966814-supplementary.pdf]

Electronic Supplementary Information's (ESI)

Figure S1. Variation of the maximum particle size versus the concentration of silane modifier.

Figure S2. AFM images of 5% silane modified coatings cured at 100, 130 and 150 °C.

Figure S3. A scheme describing the four steps of the preparation of the reference hybrid sol-gel material by MacHugh, et al., 2019 [29].

Table S1. Material formulations for functionalised alkoxysilane at various concentrations (1.0-20 mol. %) against the methoxy groups of the MAPTMS.

Figure S1. Variation of the maximum particle size versus the concentration of silane modifier.

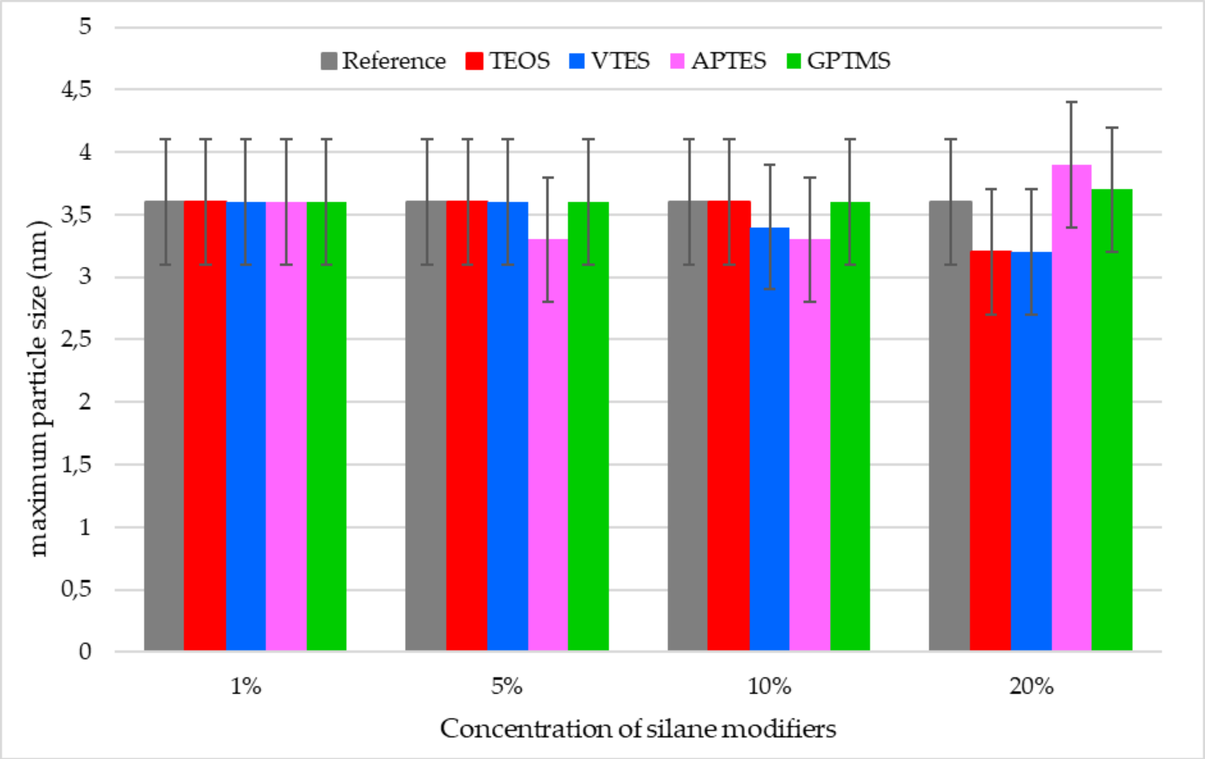

Figure S2. AFM images of 5% silane modified coatings cured at 100, 130 and 150 °C.

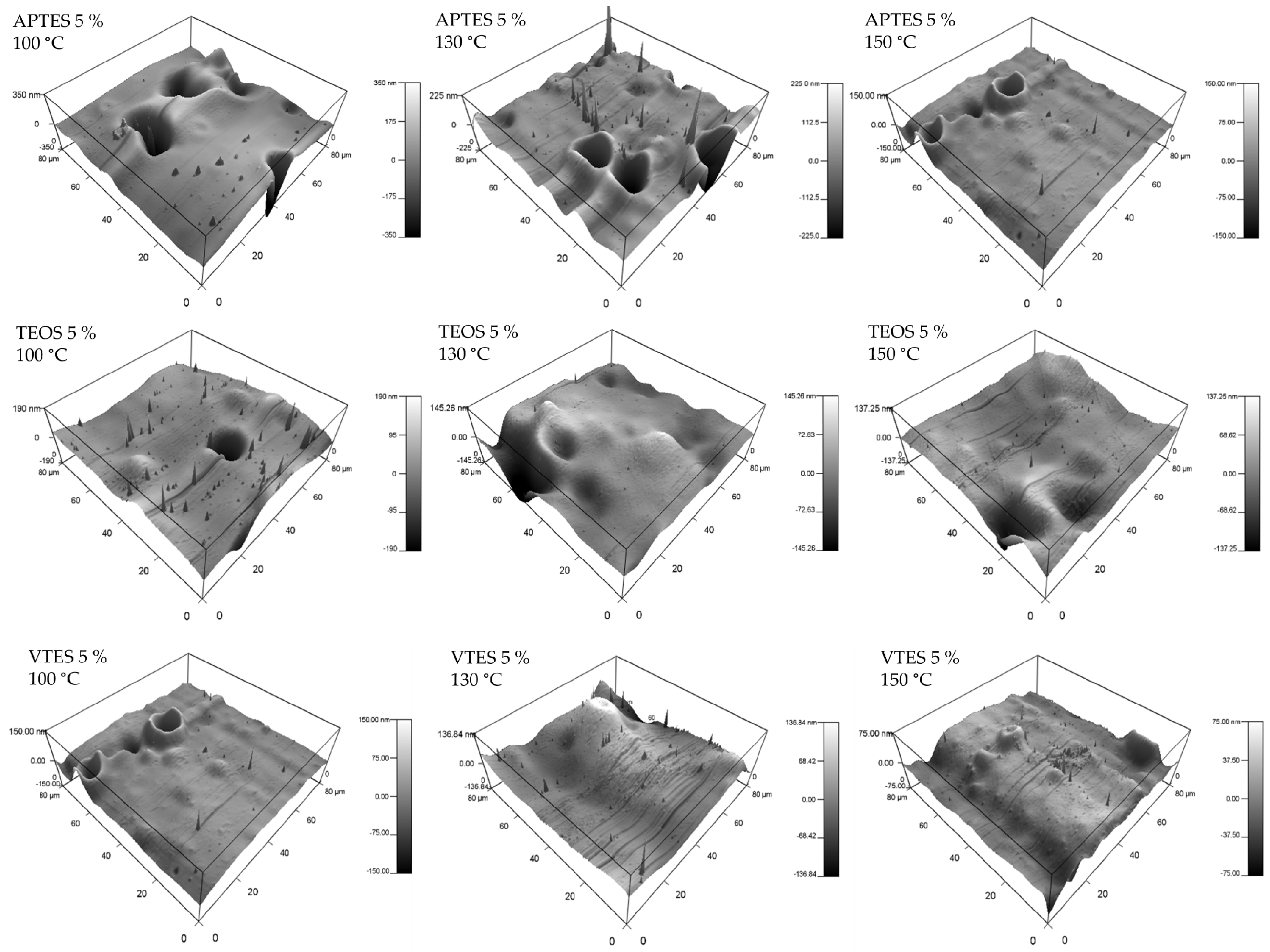

Figure S3. A scheme describing the four steps of the preparation of the reference hybrid sol-gel material by MacHugh, et al., 2019 [29].

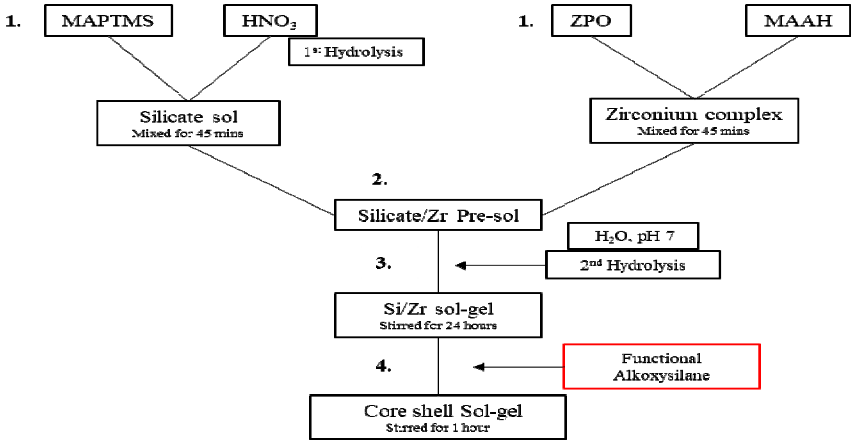

Table S1. Material formulations for functionalised alkoxysilane at various concentrations (1.0-20 mol. %) against the methoxy groups of the MAPTMS

Functional Alkoxysilane	Concentration (mol. %)
APTES	1
APTES	5
APTES	10
APTES	20
GPTMS	1
GPTMS	5
GPTMS	10
GPTMS	20
TEOS	1
TEOS	5
TEOS	10
TEOS	20
VTES	1
VTES	5
VTES	10
VTES	20
